# The Effects of Moderate Alcohol Consumption on Circulating Metabolites and Gut Microbiota in Patients With Coronary Artery Disease

**DOI:** 10.3389/fcvm.2021.767692

**Published:** 2021-11-02

**Authors:** Xinyue Zhao, Ruilin Zhou, Hanyu Li, Yue Fan, Yueshen Sun, Xiaomin Hu, Shuyang Zhang

**Affiliations:** ^1^State Key Laboratory of Complex Severe and Rare Diseases, Department of Cardiology, Peking Union Medical College Hospital, Chinese Academy of Medical Science & Peking Union Medical College, Beijing, China; ^2^Department of Medical Research Center, Peking Union Medical College Hospital, Chinese Academy of Medical Science & Peking Union Medical College, Beijing, China

**Keywords:** coronary artery disease, alcohol, serum metabolites, gut microbiome, multi-omics

## Abstract

**Background:** Epidemiological studies confirmed that moderate alcohol consumption was associated with a reduced risk of adverse cardiovascular events. It is increasingly recognized that the composition of gut microbiota and metabolites is involved in modulating the cardiovascular health of the host. However, the association of moderate alcohol consumption with serum metabolites and gut microbiome and its impact on coronary artery disease (CAD) is not fully investigated.

**Method:** Serum untargeted metabolomics analysis and fecal 16S rRNA sequencing were performed on 72 male patients with CAD having various alcohol consumption (36 non-drinkers, 18 moderate drinkers, and 18 heavy drinkers) and 17 matched healthy controls. MetaboAnalyst and PICRUSt2 were utilized to analyze the possible involved metabolic pathways. Multi-omics analysis was achieved by Spearman correlation to reveal the interactions of alcohol consumption with gut microbiome and serum metabolites in patients with CAD.

**Results:** We noted distinct differences between patients with CAD, with varying levels of alcohol consumption and healthy controls in aspects of serum metabolome and the gut microbiome. Moderate alcohol consumption significantly changed the lipidomic profiles, including reductions of sphingolipids and glycerophospholipids in moderate drinkers with CAD when compared with non and heavy drinkers with CAD. Moreover, we also found the reduction of microbial-derived metabolites in moderate drinkers with CAD, such as 2-phenylacetamide and mevalonic acid. To be noted, the gut microbiota of moderate drinkers with CAD tended to resemble that of healthy controls. Compared with non-drinkers, the relative abundance of genus *Paraprevotella, Lysinibacillus* was significantly elevated in moderate drinkers with CAD, while the genus *Bifidobacterium, Megasphaera*, and *Streptococcus* were significantly reduced in moderate drinkers with CAD. Multi-omics analysis revealed that specific metabolites and microbes associated with moderate alcohol consumption were correlated with the severity of CAD.

**Conclusions:** Our study revealed that the impact of moderate alcohol consumption on serum metabolites and gut microbiota in patients with CAD seemed to be separated from that of heavy and non-alcohol consumption. Moderate drinking tended to have more positive effects on metabolic profiles and commensal flora, which may explain its beneficial effects on cardiovascular health. Overall, our study provides a novel insight into the effects of moderate alcohol consumption in patients with CAD.

## Introduction

Coronary artery disease is one of the most common causes of death in the general population (1), and alcohol abuse is a widely recognized risk factor in adverse cardiovascular events. Alcohol affects the cardiovascular system of human beings in several ways. It was reported that alcohol abuse can be associated with various adverse cardiovascular events, such as stroke, acute heart failure, and sudden cardiac death (2). However, clinical studies demonstrated that moderate drinking presented an inverse correlation with the incidence of coronary artery disease, perhaps due to its effects on lipoprotein levels (3). Epidemiological studies illustrated that moderate alcohol intake (one to two cups a day, or 100 grams per week) was associated with a decreased incidence of CAD (4, 5). What is more, the association between the severity of cardiovascular diseases and different alcohol consumption presented a *U*-shaped curve. In other words, light to moderate alcohol intake had a reduced risk of cardiovascular events, while the relationship reversed in chronic alcohol overconsumption (4–8). In addition, recent studies have reported possible mechanisms for the protective effects of moderate alcohol consumption on patients with CAD. Some research reported that moderate alcohol consumption may elevate the level of high-density lipoprotein cholesterol (HDL-C), apolipoprotein A, and adiponectin levels (9, 10), which were considered as protective factors in cardiovascular disease. It was also reported that moderate alcohol consumption may affect the modulation of insulin resistance and chronic inflammation. By suppressing inflammation and promoting vasodilation, adverse cardiovascular events can be reduced (11). Furthermore, by moderate alcohol consumption, the hypercoagulable state of patients with CAD may be reduced by the anticoagulation effect of alcohol (12). Overall, it was believed that moderate alcohol consumption was associated with a reduced risk of cardiovascular events by various possible mechanisms.

Meanwhile, it is increasingly recognized that the composition of gut microbiota and serum metabolites is involved in modulating the cardiovascular health of the host. In recent years, more and more studies have confirmed that alterations in the gut microbiome were associated with the severity of CAD (13–15). Gut microbiota participates in modulating the cardiovascular health of the host through its metabolites, such as short-chain fatty acids (SCFA), trimethylamine N-oxide (TMAO), and phenylacetylglutamine (PAGln) (16–18). Alterations in serum metabolome, such as C34:2 hydroxy-phosphatidylcholine, N-acetylneuraminic acid, sphingomyelin (SM), were reported to be significantly associated with CAD and were considered as potential biomarkers (19–22). Moreover, the metabolic features of alcohol drinkers were investigated and showed profound modifications of lipidomic profiles and amino acids in humans (23–25). Alcohol use was associated with lower levels of phosphatidylcholine acyl-alkyls, hydroxy-sphingomyelin, glutamine, and citrate, and higher concentration of fatty acids, phosphatidylcholine diacyls, tyrosine, and alanine (23). Many metabolic profiles showed U-shape associations with alcohol consumption, such as total triglycerides and phenylalanine (24). Thus, there may exist the possibility that moderate alcohol consumption may have a protective effect on cardiovascular health by modulating the gut microbiome and serum metabolome.

To address the question above, we recruited a total of 89 patients with CAD enrolled at Peking Union Medical College Hospital (PUMCH) and divided them into four groups (detailed in the “Materials and Methods” section): healthy controls (HC) (*n* = 17), non-drinkers with CAD (ND-CAD) (*n* = 36), moderate drinkers with CAD (MD-CAD) (*n* = 18), and heavy drinkers with CAD (HD-CAD) (*n* = 18). Detailed clinical data of all 89 subjects were collected. Moreover, 16S rRNA sequencing and liquid chromatography-mass spectrometry (LC-MS) were applied. The aim of this study was to reveal the association between moderate drinking and gut microbiome, serum metabolome in patients with CAD, as well as key alterations related to clinical benefits.

## Materials and Methods

### Study Population

The participants in the study were recruited consecutively at the department of cardiology in Peking Union Medical College Hospital from 2016 to 2018. The inclusion criteria were as follows: (1) male patients (to exclude the influence of hormone); (2) patients who exhibited ≥ 50% stenosis in at least one main coronary artery in coronary angiography; exclusion criteria included the complication of gastrointestinal diseases, malignant tumor, autoimmune disorders, infectious diseases, renal dysfunction (creatine > 3 mg/dl), a history of gastrointestinal surgery within a year, and had antibiotics over 3 days in the last 3 months. In addition, the study focused on current drinkers and current non-drinkers, excluding the abstinent drinkers, who may interfere with the results. The subjects enrolled in the study had detailed questionnaires about their demographic features, living habits, and clinical information. Peripheral venous blood and stool samples were collected the next morning after admission. The preparation and the storage of blood and stool samples were described in our previous study (26). The freshly collected samples from each participant were immediately transported to our laboratory and stored in a −80°C refrigerator.

The level of alcohol consumption was assessed based on the information collected in the questionnaire, including drinking history, frequency, amount, and the most often consumed type of alcoholic beverage. The alcohol intake (g/d) was obtained by calculating drinking frequency, amount, ethanol density (0.8 g/L), and the alcohol content of each beverage (%v/v): 50.0% for liquor, 12.9% for wine, and 5.3% for beer (27). Based on the cut-off value for different drinking categories in other research (28–30), 72 patients with CAD were divided into three groups: (1) non-drinkers with CAD (ND-CAD): patients with CAD who denied a drinking history; (2) moderate drinkers with CAD (MD-CAD): patients with CAD who had an ongoing regular light-to-moderate drinking habit (the average alcohol intake was 0–40 g pure alcohol per day); (3) heavy drinkers with CAD (HD-CAD): patients with CAD who had alcoholism habits or chronic alcohol over-intake (more than 40 g pure alcohol per day).

In addition, 17 healthy volunteers who met the following criteria were enrolled as the healthy control (HC): (1) male, (2) did not suffer from CAD, (3) and did not meet any of the exclusion criteria above.

The statistical analysis of the characteristics in the study population was described in detail in our previous work (31). The study complied with the principles of the Declaration of Helsinki. All the participants in the study provided written informed consent.

### Untargeted Metabolomics Analysis

Serum metabolome analysis was conducted on a Waters ACQUITY ultra-high-performance liquid chromatography system (Milford, MA), coupled with a Waters Q-TOF Micromass system (Manchester, UK) in positive and negative ionization modes. Polar ionic and lipid modes were performed based on the properties of metabolites. Sample preparation and LC-MS experiment procedure were detailed and described previously (26). The peak-ion intensity matrix was then filtered by removing peaks with a zero value in more than 80% of samples. The threshold of the coefficient variation value of quality control samples was set at 30%. Wilcoxon rank-sum test was adopted to identify the metabolites with significant differences between groups. Partial least squares discriminant analysis (PLS-DA) was conducted by SIMCA software (v14.1, Umetrics, Sweden). Variable importance in the projection (VIP)-value > 1 and *P* < 0.05 was adopted for select important peaks. Online databases Human Metabolome Database (https://hmdb.ca), LipidMaps (https://www.lipidmaps.org), and PubChem (https://pubchem.ncbi.nlm.nih.gov/) were used for classifying peaks according to the molecular mass data (m/z). MetaboAnalyst (https://www.metaboanalyst.ca) was used to conduct pathway enrichment analysis. Pathways were regarded as potential targets with a threshold of impact-value > 0.10 when utilizing MetaboAnalyst (32).

### 16S rRNA Gene V3–V4 Region Sequencing of Fecal Microbiota and Data Analysis

Microbial DNAs were extracted from the stool samples using the bead-beating method (33). Then, the amplification of the V3–V4 region of 16S rRNA genes was performed using PCR (34). The sequencing library was established as described previously (35), and purified products were sequenced with the Illumina Miseq system (Illumina Inc., USA). The downstream amplicon analysis was conducted by EasyAmplicon v1.0 (36). Dereplication was performed by the *-derep_fullength* command of VSEARCH (v2.15) (37). Operational taxonomic units (OTU) were clustered *via* the *-cluster_otus* command of USEARCH (v10.0) at the cutoff of 97% (38). The feature table was created with *vsearch—usearch_global*. Taxonomic annotation was generated according to Greengenes database using *usearch–otutab* (39).

The sequences of all samples were downsized to the sample with the least sequences to calculate the diversity indices. Alpha diversity was evaluated with Shannon's index and Chao1 index. Beta diversity was performed by principal coordinate analysis (PCoA) and constrained PCoA (CPCoA) with Bray-Curtis distances. The composition of each group was represented as a boxplot plot at the phylum level and as a Chord diagram at the genus level with R package ggplot2. As for the comparison of differences, edgeR was applied to identify the differences between groups and the Benjamini-Hochberg method to control the FDR (40). A threshold of *P* < 0.05 with FDR < 0.2 was considered statistically significant. Bugbase and Phylogenetic Investigation of Communities by Reconstruction of Unobserved States 2 (PICRUSt2) were utilized to perform functional predication of the gut microbiota (41, 42). Furthermore, PICRUSt2 was utilized to predict the metagenomic pathways based on the MetaCyc database (43). Pathways that were significantly different between the moderate drinkers and non-drinkers were identified by Welch's *t*-test, and Storey FDR was utilized for multiple pathways. STAMP software (v2.1.3) was applied for statistical analysis and visualization of the identified pathways.

### Identification of the Key Metabolites or OTUs Associated With Moderate Alcohol Consumption

For metabolomics analysis, Wilcoxon rank-sum test was conducted, and the threshold of VIP > 1 and *P* < 0.05 was used to identify differential metabolites. To be detailed, the metabolites that were significantly higher or lower in MD-CAD than those in both ND-CAD and HD-CAD groups were defined as key metabolites associated with moderate alcohol consumption. In terms of the microbiota, using edgeR with a threshold of *P* < 0.05 and FDR < 0.2, there were a total of 21 differential OTUs between MD-CAD and ND-CAD. Among the 21 OTUs, key OTUs were defined as those whose average relative abundances represented a U-shaped or convex-shaped relationship with different levels of alcohol consumption (i.e., the key OTUs present either the highest or the lowest average relative abundance in MD-CAD when comparing with ND-CAD and HD-CAD).

### Multi-Omics Correlation Study

Spearman correlation analysis was conducted between key bacterial taxa, metagenomic pathway, serum metabolites, and clinical parameters with SPSS (v. 24.0), and visualized as a heatmap using the R package pheatmap. A Sankey plot was drawn by R package networkD3 to illustrate the interrelationship among the differential OTUs, metabolic features, and clinical parameters.

### Statistical Analysis and Visualization

Continuous normally distributed data among three groups were analyzed by one-way ANOVA. Kruskal-Wallis H-test was employed to analyze continuous data with non-normal distribution among three groups, and Mann—Whitney U test was applied for that between two groups. Categorical variables were analyzed by χ2 test or Fisher's exact test. Data were analyzed using SPSS (v.24.0). Figures were made by utilizing R 4.0.4.

## Results

### Clinical Characteristics of the Study Population

The clinical characteristics of the study cohort were summarized in Table 1. In general, among different alcohol consumption groups, there was no significant difference in age, body mass index (BMI), and systolic blood pressure. In terms of alcohol consumption, there were significant differences between the three disease groups. The amount of alcohol intake was significantly higher in HD-CAD when compared with MD-CAD (*P* < 0.001), and liquor was the most common alcohol intake type. Drinking history, drinking years, and drinking frequency showed no significant difference. The three disease groups showed no significant difference in hypertension (HTN), type 2 diabetes mellitus (T2DM), hyperlipidemia (HLP), and fatty liver disease (FLD). In terms of CAD severity, although there was no significant difference in Gensini score. Gensini score is an assessment of the CAD severity using coronary angiography and positively associated with disease severity (44). The average Gensini score of MD-CAD [27.50 (16.00, 42.75)] was the lowest when compared with ND-CAD [39.50 (19.25, 66.75)] and HD-CAD [34.75 (24.75, 56.00)]. Also, the average level of CK-MB [CK-MB is a cardiac biomarker that represents cardiovascular injury (45)] in MD-CAD was the lowest when compared with non-drinkers and heavy drinkers, so as the level of hs-CRP [The level of hs-CRP represents the inflammation status in the human body (46), and chronic inflammation plays a central role in the development of cardiovascular disease (47)]. The other laboratory tests, such as total cholesterol (TC), triacylglycerol (TG), low-density lipoprotein cholesterol (LDL-C), HDL-C, alanine aminotransferase (ALT), and aspartate aminotransferase (AST), showed no significant difference among the three disease groups. Thus, moderate alcohol consumption was associated with the improvement of CAD severity, that is, MD-CAD presented relatively better status than other patients with CAD.

**Table 1:** Characteristics of the study population.

**Variable**	**HC** **(***n*** = 17)**	**ND-CAD** **(***n*** = 36)**	**MD-CAD** **(***n*** = 18)**	**HD-CAD** **(***n*** = 18)**	***P-*** **Value**
**Demographics**					
Age, years^*^	57.7 ± 12.2	65.2 ± 11.1	60.2 ± 10.5	58.9 ± 8.3	0.058
BMI, kg/m^2^^*^	24.85 ± 3.01	25.50 ± 2.86	25.72 ± 3.51	26.19 ± 1.96	0.582
SBP, mmHg^#^	120.00 (113.00, 130.00)	130.00 (118.25, 137.00)	133.50 (123.00, 139.25)	127.00 (112.75, 134.50)	0.061^a^
**Type of CAD**					
SCAD^†^	NA	13 (36.1)	5 (27.8)	5 (27.8)	NA
UA^†^	NA	19 (52.8)	10 (55.6)	7 (38.9)	NA
MI^†^	NA	4 (11.1)	3 (16.7)	6 (33.3)	NA
Gensini^#^	NA	39.50 (19.25, 66.75)	27.50 (16.00, 42.75)	34.75 (24.75, 56.00)	0.613
**Drinking information**					
Drink history^†^	5 (29.4)	0 (0)	18 (100)	18 (100)	<0.001^b^, ^c^
Drink years^#^	NA	NA	30.0 (18.8, 40.0)	27.5 (20.0, 40.0)	0.736
Frequency, per week^#^	NA	–	7 (1,7)	7 (7,7)	0.203
Types^†^	NA	NA	Liquor: 16 (88.9) Wine: 1 (5.6) Beer: 1 (5.6)	Liquor: 18 (100) Wine: 0 (0) Beer: 0 (0)	NA
Alcohol intake, g/d^#^	NA	NA	21.4 (5.4, 25.0)	87.5 (60.3, 131.3)	<0.001^d^
**Medications**					
HTN, %^†^	5 (29.4)	20 (55.6)	11 (61.1)	15 (83.3)	0.036^a^
T2DM, %^†^	1 (5.9)	13 (36.1)	4 (22.2)	7 (38.9)	0.070^a^
HLP, %^†^	5 (29.4)	22 (61.1)	9 (50.0)	13 (72.2)	0.065^a^
FLD, %^†^	4 (23.5)	5 (13.9)	5 (27.8)	3 (16.7)	0.593
Statin, %^†^	1 (5.9)	12 (33.3)	5 (27.8)	9 (50.0)	0.031^a^
HTN-Drugs, %^†^	5 (29.4)	20 (55.6)	10 (55.6)	16 (88.9)	0.010^a^, ^c^, ^d^
OAD, %^†^	1 (5.9)	6 (16.7)	2 (11.1)	7 (38.9)	0.086
**Laboratory test**					
TC, mmol/L^*^	4.34 ± 0.68	3.93 ± 1.25	3.79 ± 1.02	3.78 ± 0.89	0.363
TG, mmol/L^#^	1.36 (0.73, 2.34)	1.17 (0.98, 1.54)	1.67 (1.06, 2.15)	1.18 (0.94, 2.55)	0.339^b^
LDL-C, mmol/L^#^	2.45 (2.03, 3.00)	2.12 (1.57, 2.81)	2.06 (1.77, 2.40)	1.98 (1.43, 2.47)	0.083^a^
HDL-C, mmol/L^*^	1.11 ± 0.28	0.96 ± 0.20	0.90 ± 0.20	0.95 ± 0.21	0.040^a^
ALT, U/mL^#^	20.00 (13.50, 33.00)	23.00 (18.25, 29.00)	20.00 (14.00, 37.75)	26.00 (18.75, 40.00)	0.333
CK, U/mL^#^	112.00 (91.00, 128.50)	105.50 (72.75, 142.00)	91.00 (75.50, 129.00)	81.50 (70.50, 103.50)	0.192
CK-MB, U/mL^#^	070 (0.50, 0.95)	0.80 (0.50, 1.75)	0.60 (0.45, 1.08)	0.70 (0.60, 1.00)	0.530
cTnI, ug/L^#^	0.00 (0.00, 0.00)	0.00 (0.00, 0.02)	0.00 (0.00, 0.01)	0.03 (0.01, 0.13)	<0.001^a^, ^c^, ^d^
Hs-CRP, mg/L^#^	0.50 (0.35, 1.01)	1.86 (0.90, 4.37)	1.08 (0.63, 4.51)	2.39 (1.80, 4.11)	0.002^a^

Data are presented with *mean ± SD,

# *median (IQR), or*

† *number (%)*.

*Continuous normally distributed data among three groups were analyzed by one-way ANOVA. Kruskal-Wallis H-test was employed to analyze continuous data with non-normal distribution among three groups, and Mann—Whitney U-test was applied for that between two groups. Categorical variables were analyzed by χ*

2 *test or Fisher's exact test. N/A, not available; SBP, systolic blood pressure; BMI, body mass index; SCAD, stable coronary artery disease; UA, unstable angina; MI, myocardial infarction; HTN, hypertension; T2DM, type 2 diabetes mellitus; HLP, hyperlipidemia; FLD, fatty liver disease; HTN drug, antihypertensive drugs; OAD, oral antidiabetic drugs; TC, total cholesterol; TG, triacylglycerol; LDL-C, low-density lipoprotein cholesterol; HDL-C, high-density lipoprotein cholesterol; ALT, alanine aminotransferase; AST, aspartate aminotransferase; CK, creatine kinase; CK-MB, creatine kinase-MB; cTnI, cardiac troponin I; HsCRP, high-sensitivity C-reactive protein*;

a *P < 0.05 for HC vs. all patients with CAD*.

b *P < 0.05 for ND-CAD vs. MD-CAD*.

c *P < 0.05 for ND-CAD vs. HD-CAD*.

d *P < 0.05 for MD-CAD vs. HD-CAD*.

### Serum Metabolome Presented Significant Alterations in Patients With CAD, With Different Alcohol Consumption

**Figure 1:**
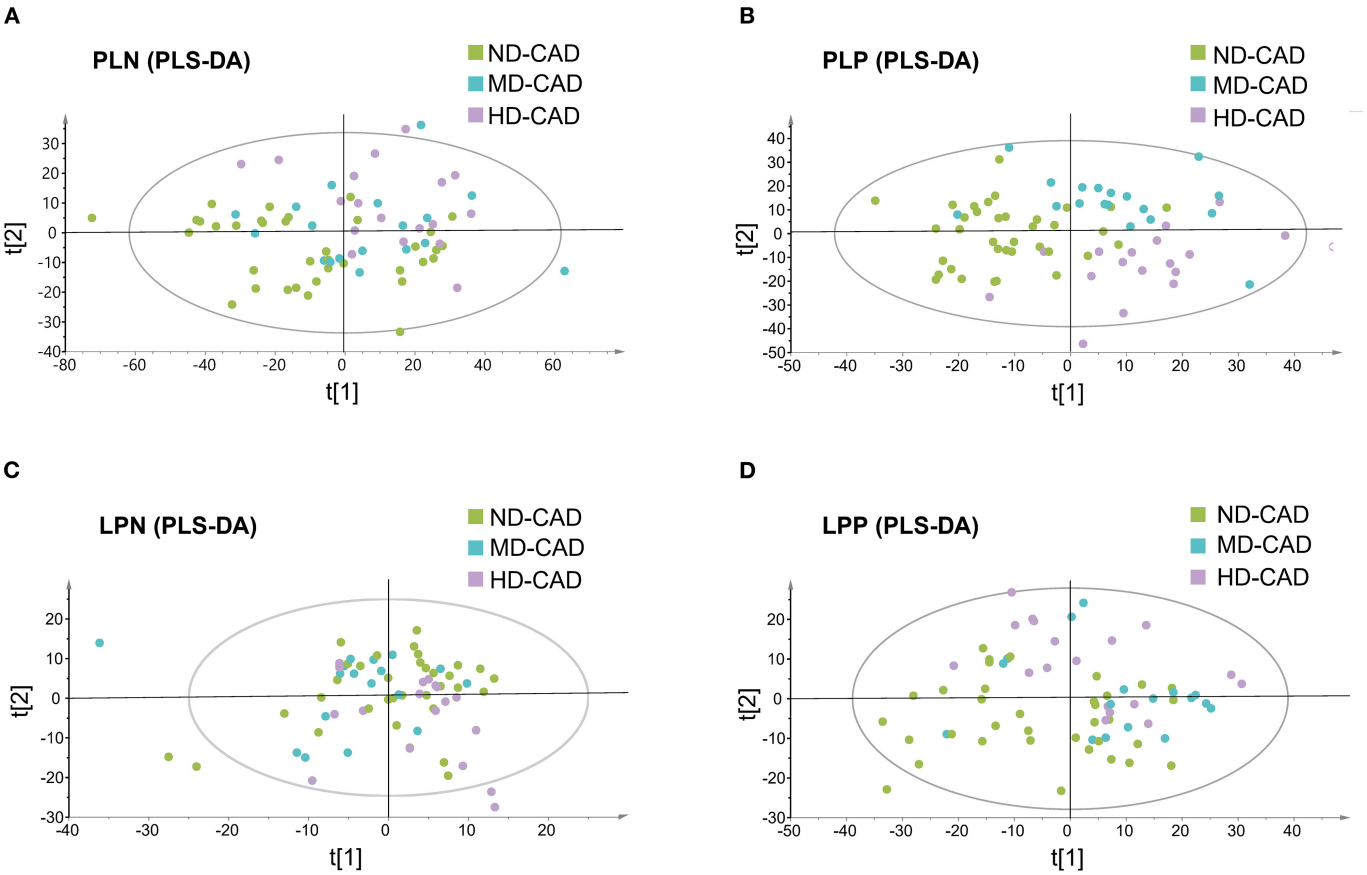
Serum metabolites profiles of the patients with coronary artery disease with different alcohol consumption. **(A–D)** Separation of serum metabolites in polar ionic negative (PLN) mode, polar ionic positive (PLP) mode, lipid negative (LPN) mode, and lipid positive (LPP) mode, revealed by PLS-DA modeling.

Since it was reported that alcohol may affect serum metabolic profiles, serum metabolome in ND-CAD, MD-CAD, and HD-CAD was explored through the untargeted LC-MS method. After QC and removal of peaks with low abundance, a total of 10,416 metabolites in polar ionic mode and 3,131 in lipid mode were obtained. PLS-DA modeling was utilized to reveal the alteration of serum metabolites. The score scatter plots of four modes were shown in Figures 1A–D, and the parameters of each mode were provided in Supplementary Table 1. It was obvious that the metabolic feature of three diseased groups (ND-CAD, MD-CAD, and HD-CAD) showed significant differences in score scatter plot, especially in polar ionic positive mode (Figure 1B) and lipid positive mode (Figure 1D).

### Serum Metabolome Alterations in MD-CAD Were Associated With Cardiovascular Health Status

**Figure 2:**
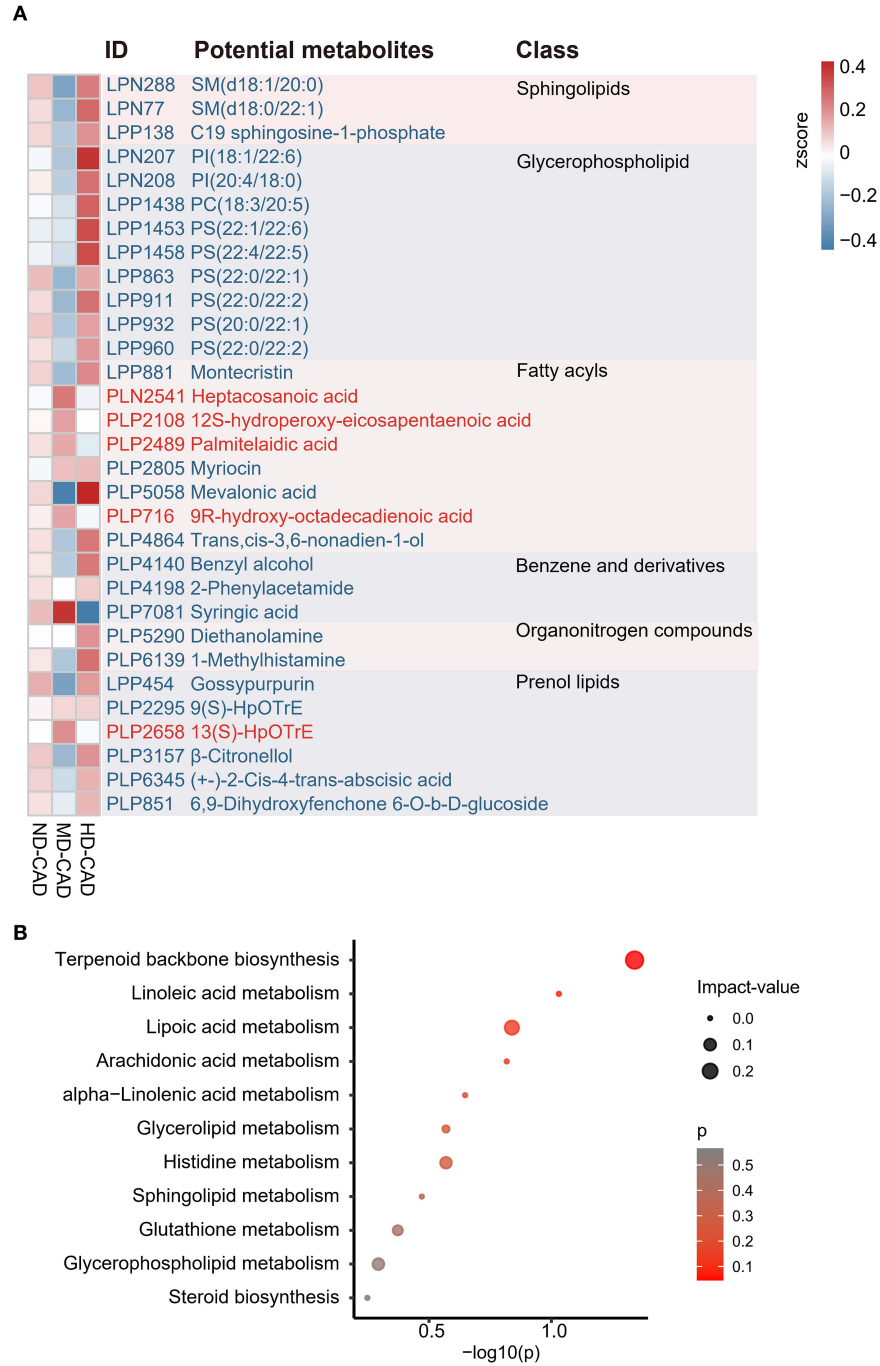
Identification of the key metabolites associated with alcohol consumption in patients with CAD. **(A)** Heatmap showing part of the key metabolites associated with moderate alcohol consumption across three groups with CAD. **(B)** Bubble map demonstrating the potential metabolic pathways associated with moderate alcohol consumption using MetaboAnalyst. The IDs and the annotated potential metabolites are highlighted in red (enriched in MD-CAD) or blue (depleted in MD-CAD).

A total of 203 serum metabolites were identified as key serum metabolites associated with moderate alcohol consumption with a threshold of VIP > 1 and Wilcoxon rank-sum *P* < 0.05 (Supplementary Table 2). All the 203 key metabolites in MD-CAD were significantly different from those in ND-CAD or HD-CAD, either the most abundant or the least in the three disease groups (Supplementary Table 2). In other words, the relative abundance of these 203 metabolites showed a “*U*” shaped or convex curve with alcohol consumption. Since MD-CAD tended to have better cardiovascular health according to the previous studies, we speculated that the most abundant metabolites (i.e., convex-shaped metabolites) in MD-CAD may have protective effects in cardiovascular health, while the most deprived metabolites (i.e., “U” shaped metabolites) may be harmful. Among these 203 metabolites, we observed unignorable quantities of metabolites were lipids and lipids-like molecules, such as sphingolipids, glycerophospholipids, prenol lipids, and fatty acyls (Figure 2A), which were consistent with other existing reports (23–25). It was reported that a high plasma level of SM was an independent risk factor in cardiovascular diseases and predicted a poor prognosis of CAD (48, 49). Consistently, we observed sphingolipids, such as SM (d18:0/22:1) (LPN77) and SM (d18:1/20:0) (LPN288), were depleted in MD-CAD when compared with the other two diseased groups. Moreover, we observed all annotated glycerophospholipid showed significant depletion in MD-CAD when compared with other two CAD groups (ND-CAD and HD-CAD) (Figure 2A), including phosphatidylinositol [PI, such as PI (18:1/22:6) (LPN207) and PI (20:4/18:0) (LPN208)], phosphatidylcholine [PC, such as PC (18:3/20:5) (LPP1438)], phosphatidylserine [PS, such as PS (22:1/22:6) (LPP 1453), PS (22:4/22:5) (LPP1458), PS (22:0/22:1) (LPP863), PS (22:0/22:2) (LPP911), PS (22:0/22:1) (LPP863), PS (20:0/22:2) (LPP960), PS (20:0/22:1) (LPP932), and PS (22:0/22:2) (LPP960)]. Since glycerophospholipid was associated with macrophage-driven inflammation (50) and atherosclerosis (51), the depletion of glycerophospholipid in MD-CAD may be associated with cardiovascular health. What is more, PC can be degraded by the gut microbiome and thus converted to TMAO, a well-known risk factor in the major adverse cardiovascular events (17, 52, 53). Thus, a higher level of PC was often associated with higher cardiovascular risk (17, 52), and (53)]. Consistently, PC was observed to be depleted in MD-CAD when compared with the other two diseased groups.

To further elucidate the cause of serum metabolome change, metabolic pathway enrichment was performed by MetaboAnalyst. The results showed that these differential metabolites mainly clustered in 11 pathways (Figure 2B, Supplementary Table 3). Among the 11 pathways, terpenoid backbone synthesis (*P* = 0.28955, impact-value = 0.0.11429), and lipoic acid metabolism (*P* = 0.14053, impact-value = 0.16667) were regarded as potential target pathways. Terpenoid had significant effects on the prevention and treatment of cardiovascular disease (54), while lipoic acid was reported to be beneficial in cardiovascular disease by reducing atherosclerosis (55, 56).

### Gut Microbiome Taxonomic Features of Moderate Drinkers With CAD Resembles That of Healthy Controls

**Figure 3:**
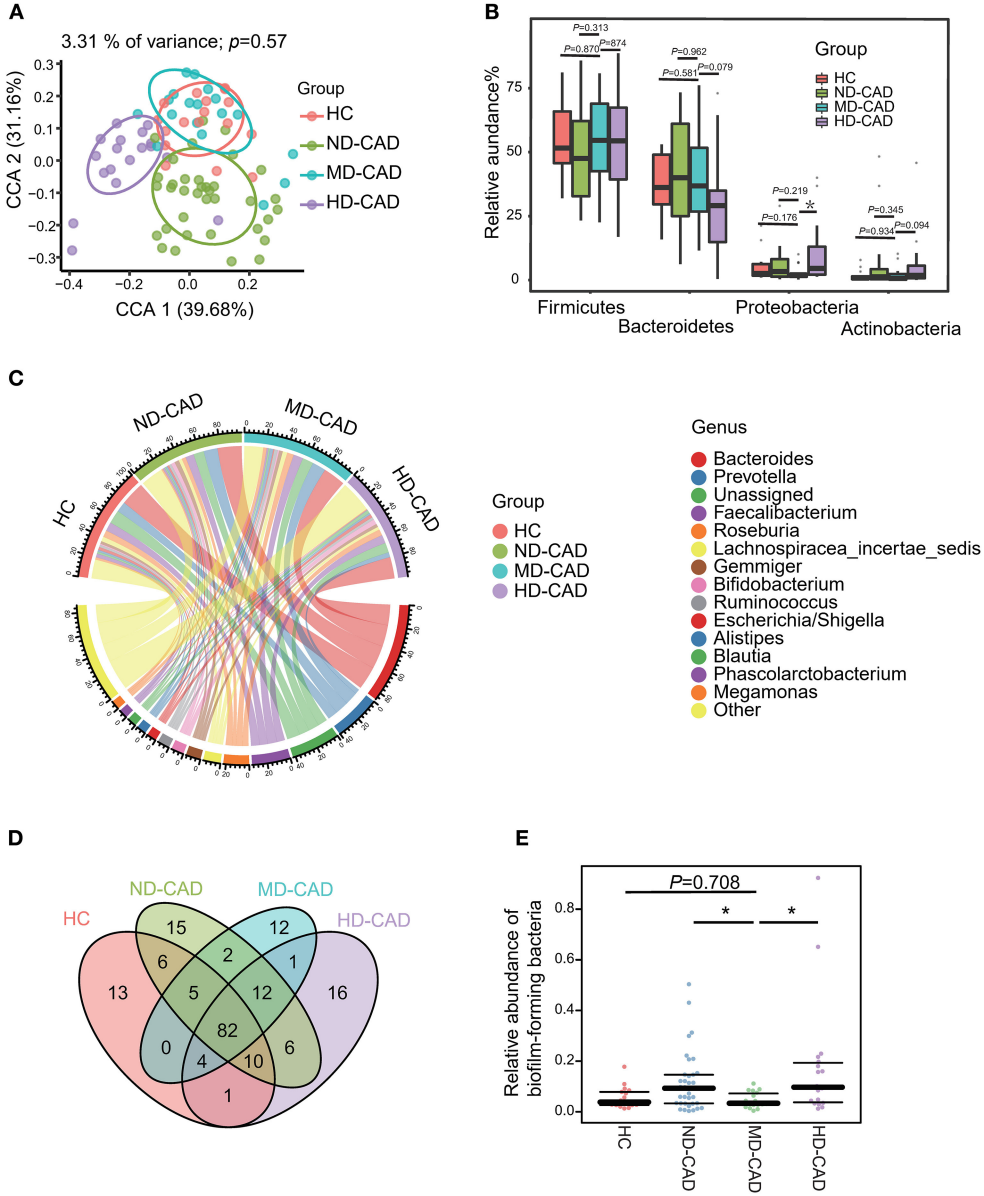
Different alcohol consumption affects the taxonomic features of gut microbiota in patients with CAD. **(A)** Beta diversity analyzed by CPCoA plot based on Bray-Curtis distances (*P* = 0.57, Adonis Test). **(B)** Comparison of the relative abundance of gut microbiota at the phylum level (ns, not significant, *P < 0.05, Mann—Whitney *U*-test. Phylum Proteobacteria: MD-CAD vs. HC: *P* = 0.176, MD-CAD vs. ND-CAD: *P* = 0.219, MD-CAD vs. HD-CAD: *P* = 0.019). **(C)** Chord plot showing the dominant genera and their contribution to each group. **(D)** Venn plot indicating the number of overlapped OTUs. **(E)** Relative abundance of biofilm-forming bacteria predicted by BugBase (ns, not significant, *P < 0.05, Mann—Whitney *U*-test. MD-CAD vs. HC: *P* = 0.708, MD-CAD vs. ND-CAD: *P* = 0.015, MD-CAD vs. HD-CAD: *P* = 0.007).

Since some of the differential metabolites may be microbiome derived, such as PC, we further elucidate the taxonomic features of the gut microbiome by 16S rRNA sequencing with sufficient depths (Supplementary Figure 1A). A total of 626 OTUs were obtained. In terms of alpha-diversity, no significant difference was observed in HC, ND-CAD, MD-CAD, and HD-CAD (Supplementary Figures 1B,C). As for beta-diversity, although no significant difference was observed (Supplementary Figure 1D) among three diseased groups in PCoA analysis, the taxonomic features of MD-CAD were almost overlapping with HC while the other two disease groups separated clearly from HC in CPCoA analysis (Figure 3A). Firmicutes and Bacteroides were the two most dominant phyla in all groups (Figure 3B, Supplementary Table 4). We noted that MD-CAD and HC presented similar proportions of Actinobacteria and Proteobacteria, while ND-CAD and HD-CAD presented a much higher proportion of the two phyla (Figure 3B). It could be inferred that the taxonomic features of the gut microbiome in MD-CAD were more similar with healthy people when compared with the other two disease groups. What is more, *Bacteroides, Prevotella*, and *Faecalibacterium* were the top three abundant genera (Figure 3C) in all four groups. Venn plot was utilized to further illustrate the taxonomic features with a relative abundance >0.1% in HC, ND-CAD, MD-CAD, and HD-CAD (Figure 3D). A total of 82 OTUs coexisted in four groups, and 94 were specifically shared by three CAD groups. Twelve OTUs were unique in MD-CAD, while those in ND-CAD and HD-CAD were 15 and 16. We further predicted the phenotypes of the gut microbiome based on their 16S rRNA sequence utilizing BugBase. Notably, HC and MD-CAD had a similar abundance of “biofilm-forming” bacteria, while ND-CAD and HD-CAD had a significantly higher abundance of “biofilm-forming” bacteria (Figure 3E). Thus, it was clear that MD-CAD presented more similar taxonomic and functional features with HC than ND-CAD and HD-CAD. This taxonomic similarity may partially explain the healthier cardiovascular status discovered in MD-CAD.

### Variations of Microbiota in MD-CAD Were Associated With Cardiovascular Health

**Figure 4:**
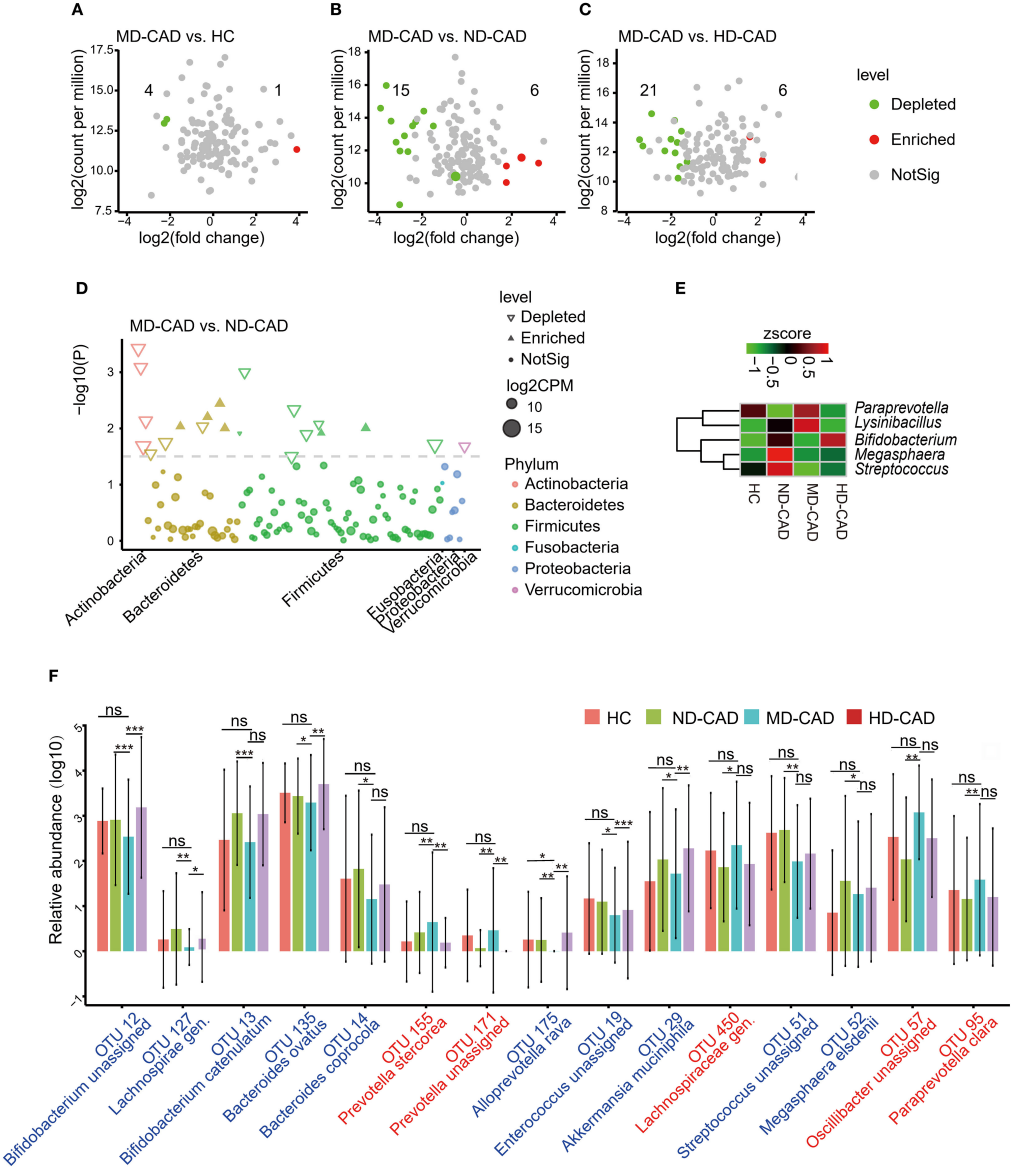
Moderate alcohol consumption was associated with certain changes of intestinal flora in patients with CAD. **(A–C)** Volcano plots showing differential OTUs in patients with CAD with different levels of alcohol consumption. **(A)** MD-CAD vs. HC. **(B)** MD-CAD vs. ND-CAD. **(C)** MD-CAD vs. HD-CAD. The numbers labeled in the figures indicated significantly depleted OTUs (highlighted in green) or enriched OTUs (highlighted in red) in MD-CAD when compared with HC, ND-CAD, and HD-CAD, respectively. **(D)** Manhattan plot demonstrating the differentially abundant OTUs and their contributions to each phylum. **(E)** Heatmap showing the relative abundance of the genera that were significantly different between MD-CAD and ND-CAD. **(F)** An overview of the relative abundance of the differential OTUs associated with moderate alcohol consumption. The differential OTUs were filtered with *P* < 0.05 as well as FDR < 0.2. ns, not significant. ^*^P < 0.05 and FDR < 0.2, ^**^P < 0.01 and FDR < 0.2, ^***^P < 0.001 and FDR < 0.2, analyzed by edgeR.

To further elucidate the alterations of the gut microbiome in MD-CAD, edgeR was utilized with a threshold of *P* < 0.05 and FDR < 0.02. Only five OTUs showed significant differences between HC and MD-CAD (Figure 4A). However, there were 21 significant differentials OTUs between MD-CAD and ND-CAD (Figure 4B), while there were 27 significant differentials OTUs between MD-CAD and HD-CAD (Figure 4C). It was obvious that, regarding MD-CAD, more differential OTUs were found when comparing with ND-CAD and HD-CAD than comparing with HC. This further confirmed the similarity of gut microbiome taxonomic features between HC and MD-CAD. We suspected that it was moderate alcohol consumption that tended to alter the gut microbiome of patients with CAD to a more similar feature with HC. We further looked into the differential microbiome between ND-CAD and MD-CAD. At the phylum level, Actinobacteria, Firmicutes, and Bacteroidetes were found significantly different (Figure 4D). At the genus level, *Paraprevotella* and *Lysinibacillus* were significantly elevated in MD-CAD while *Bifidobacterium, Megasphaera*, and *Streptococcus* were significantly depleted in MD-CAD when compared with ND-CAD (Figure 4E). At the species level, we paid attention to 15 key OTUs associated with moderate alcohol consumption (key OTUs were defined in the “Materials and Methods” section) that showed significant differences between MD-CAD and ND-CAD (Figure 4F, Supplementary Table 5). *Bacteroides ovatus* (OTU 135, depleted in MD-CAD) was found to drive the production of IgA (57), an autoimmune disease inflammatory mediator (58), and was thus regarded as a driver of cardiovascular diseases. *Prevotella stercorea* (OTU 155, enriched in MD-CAD) was reported to be beneficial in overweight patients (59). *Bacteroides coprocola* (OTU 14, depleted in MD-CAD) was reported to be enriched in patients with hypertension (60). Our result indicated that potentially beneficial bacteria were possibly promoted, and potentially pathogenic bacteria were possibly suppressed by moderate alcohol consumption.

In addition, we predicted possible functional pathways by utilizing PICRUSt2. We found 12 significantly affected pathways in MD-CAD when compared with ND-CAD (Supplementary Figure 2, Supplementary Table 6). Moreover, we found that the microbes depleted in MD-CAD (OTU 19, OTU 13, OTU 12, OTU 51, and OTU 135) were all positively correlated with pathways that were depleted in MD-CAD, especially the methionine-related pathways [superpathway of L-methionine biosynthesis (transsulfuration), L-methionine biosynthesis I, and superpathway of S-adenosyl-L-methionine biosynthesis]. In addition, we found most of these pathways were closely associated with metabolites degradation or biosynthesis, such as glucose and glucose-1-phosphate degradation, lactose, and galactose degradation I. Thus, we inferred that there may be exhibited functional changes of the gut microbiome that led to the alterations of serum metabolome in MD-CAD.

### Multi-Omics Analysis Revealed the Association Between Alcohol Consumption, Gut Microbiome, and Serum Metabolome

**Figure 5:**
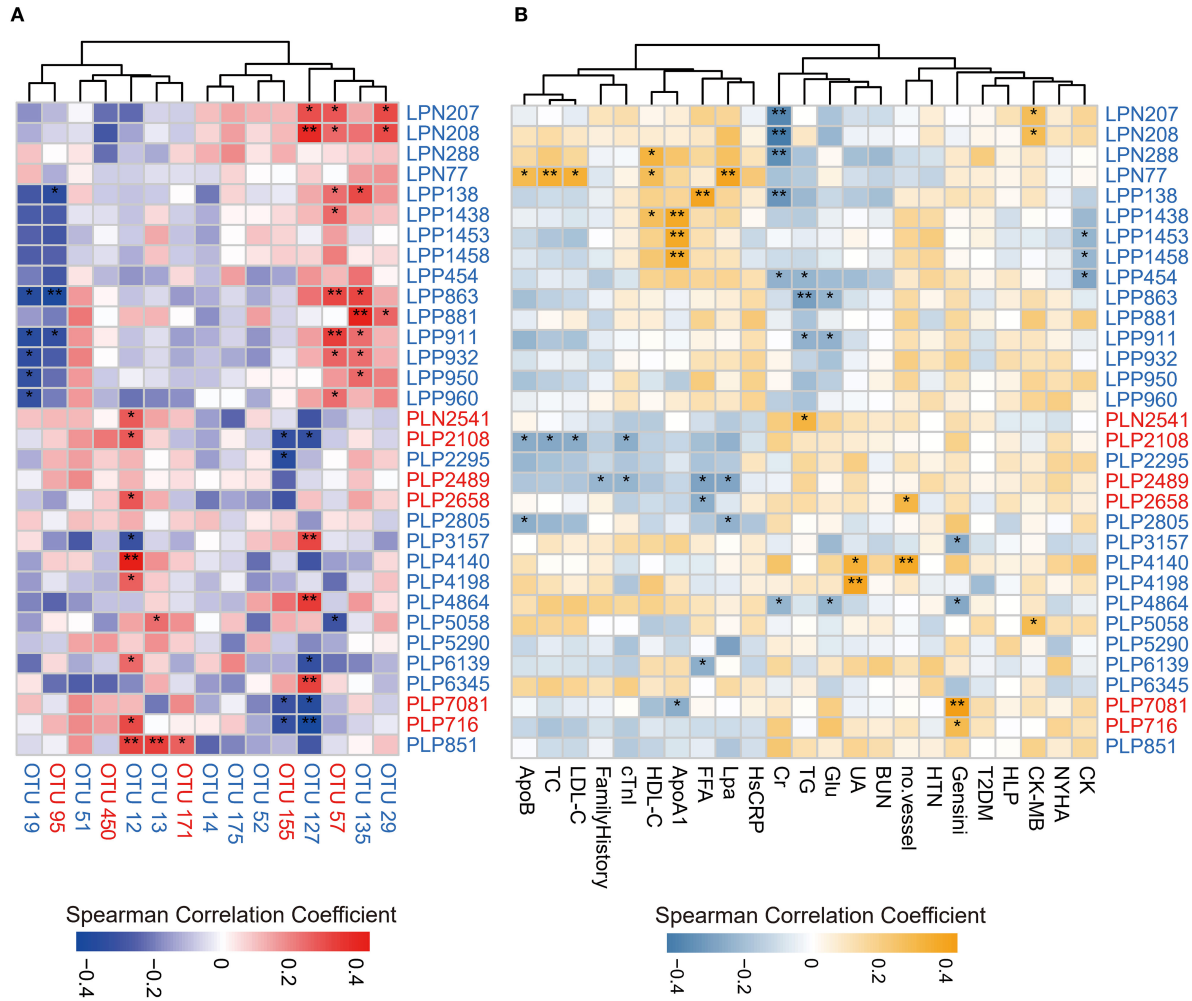
Spearman correlations between differential serum metabolites and OTUs associated with moderate alcohol consumption or major clinical parameters for CAD. **(A)** Spearman correlations between differential serum metabolites and OTUs associated with moderate alcohol consumption. **(B)** Spearman correlations between differential serum metabolites and major clinical parameters associated with moderate alcohol consumption. The IDs of metabolites or OTUs are highlighted in red (enriched in MD-CAD) and blue (depleted in MD-CAD). ^*^P < 0.05, ^**^P < 0.01. ApoB, apolipoprotein B; TC, total cholesterol; LDL-C, low-density lipoprotein cholesterol; family history: having a family history of CAD; cTnI, cardiac tropinin I; HDL-C, high-density lipoprotein cholesterol; ApoA1, apolipoprotein A1; FFA, free fatty acid; Lpa, lipoprotein (a); HsCRP, high-sensitivity C-reactive protein; Cr, creatine; TG, triglyceride; Glu, fasting blood glucose; UA, uric acid; BUN, blood urea nitrogen; no. vessel, number of stenosed vessels; HTN, hypertension; T2DM, type 2 diabetes mellitus; HLP, hyperlipidemia; CK-MB, creatine kinase-MB; CK, creatine kinase; NYHA, grade of NYHA classification.

**Figure 6:**
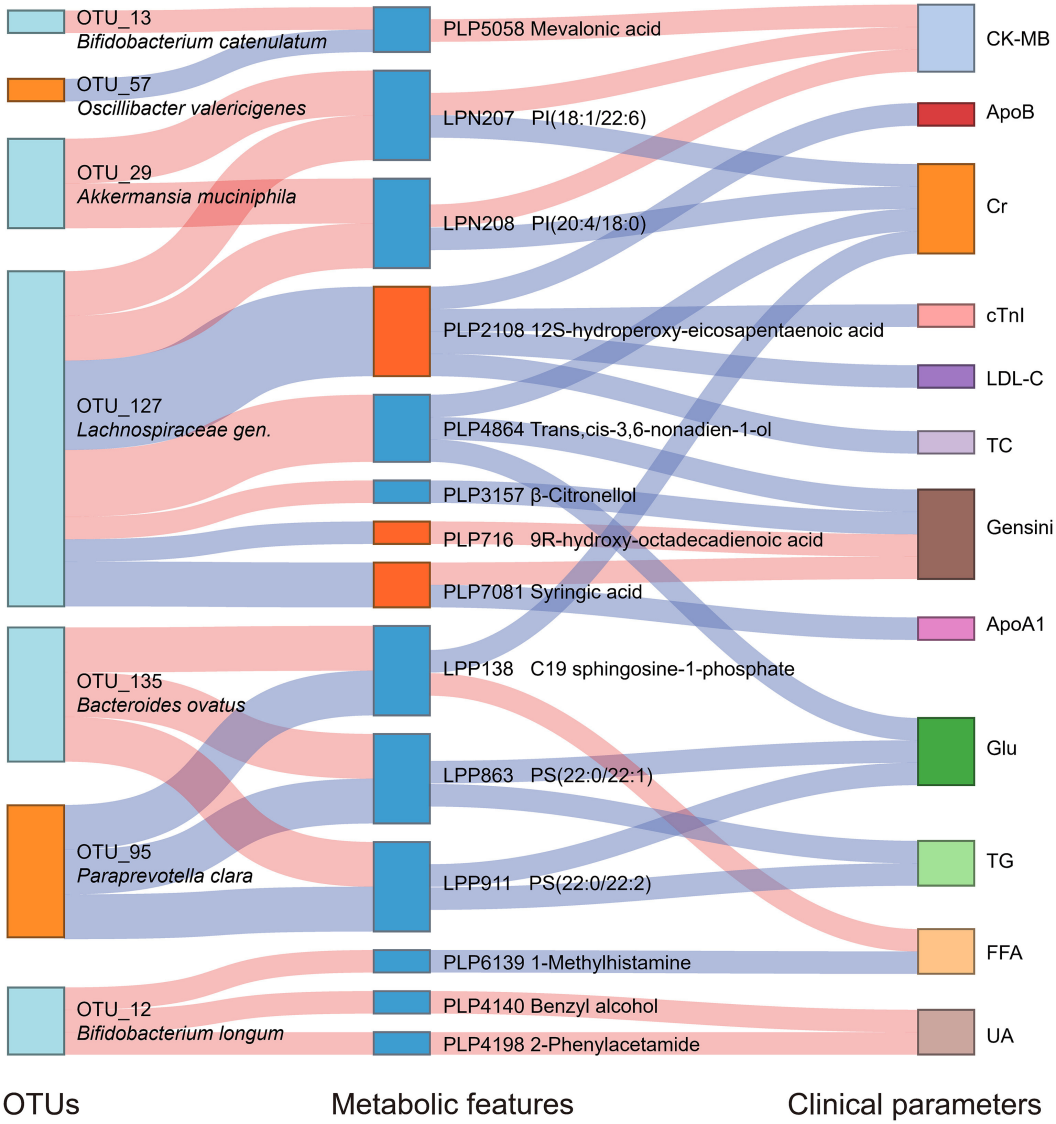
Interrelationship between intestinal flora, serum metabolic features, and major clinical parameters associated with moderate alcohol consumption with CAD. A Sankey plot was utilized to examine the relationship among the differential OTUs and serum metabolites associated with moderate alcohol consumption, and major clinical parameters of CAD. Red connections indicate significant positive correlations, and blue connections indicate significant negative correlations (Spearman correlation analysis, *P* < 0.05). In the left column, light blue boxes indicate OTUs that are significantly depleted in MD-CAD when compared with ND-CAD and HD-CAD, and light orange boxes indicate OTUs that are significantly elevated in MD-CAD when compared with ND-CAD and HD-CAD. In the middle column, dark blue boxes indicate metabolic features that are significantly decreased in MD-CAD when compared with ND-CAD and HD-CAD, and dark orange boxes indicate metabolic features that are significantly increased in MD-CAD when compared with ND-CAD and HD-CAD. The right column exhibits some major clinical parameters of CAD. CK-MB, creatine kinase-MB; cTnI, cardiac tropinin I; Cr, creatine; ApoA1, apolipoprotein A1; Glu, fasting blood glucose; TG, triglyceride; FFA, free fatty acid; UA, uric acid; ApoB, apolipoprotein B; LDL-C, low-density lipoprotein cholesterol; TC, total cholesterol.

We analyzed the association between key OTUs and serum metabolites by utilizing the Spearman correlation (Figure 5A). Several key OTUs were significantly associated with serum metabolites. We inferred that it may be the alterations in the gut microbiome that may affect the serum metabolites features. Furthermore, we correlated the key metabolites with clinical indexes and found several significant Spearman correlations (Figure 5B). We represented these interrelationships in a Sankey map, shown in Figure 6. We noted that *Bifidobacterium longum* (OTU 12, depleted in MD-CAD), *Lachnospiraceae gen*. (OTU 127, depleted in MD-CAD), and *Oscillibacter valericigenes* (OTU 57, enriched in MD-CAD) were the three main species that impacted the serum metabolome. In terms of clinical indexes, Gensini score was positively correlated with 9R-hydroxy-octadecadienoic acid (PLP716, elevated in MD-CAD) and syringic acid (PLP7081, elevated in MD-CAD), and negatively correlated with β-citronellol (PLP3157, depleted in MD-CAD) and trans,cis-3,6-nonadien-1-ol (PLP4864, depleted in MD-CAD). Moreover, cTnI negatively correlated with 12S-hydroperoxy-eicosapentaenoic acid (12-HPEPE, PLP2108, elevated in MD-CAD). Correlation analysis showed that the relative better cardiovascular health of MD-CAD may be associated with serum metabolome and gut microbiome alterations.

## Discussion

Alcohol presented complex effects on the cardiovascular system that vary with dose (61). Observational and prospective studies consistently showed a lower risk of cardiovascular and all-cause mortality in people with low levels of alcohol consumption when compared with people with alcohol abuse and non-drinkers (62). In our study, we found patients with CAD with moderate alcohol consumption presented a relatively healthier cardiovascular status, that is, a lower level of Gensini score, CK-MB, and hs-CRP. Thus, we looked further into the serum metabolome profiles and gut microbiome taxonomic features to reveal the possible associations between moderate alcohol consumption and healthier cardiovascular status.

Serum metabolome presented significant alterations in MD-CAD when compared with ND-CAD and HD-CAD. The representative metabolites were sphingolipids and glycerophospholipids, which were both significantly depleted in MD-CAD. Sphingolipids accumulate in the formation of atherosclerotic plaque both in humans and primates (63, 64). Emerging studies uncovered that a high plasma level of sphingolipids was an independent risk factor in CAD (48, 49). Moreover, studies on animal models confirmed that sphingolipids contributed to the development of obesity and insulin resistance (65). The medication targeting sphingolipid metabolism may improve the condition and prognosis of metabolic disorders (66). Consistently, in our study, the depletion of sphingolipids associated with moderate alcohol consumption tended to relieve cardiovascular stress. In addition, C19 sphingosine-1-phosphate (S1P, LPP138, depleted in MD-CAD) was reported to contribute to the pathogenesis of multiple cardiovascular disorders (67) by activating the proliferation and migration of vascular smooth muscle cells (68). Hence, we suspected that moderate alcohol consumption may suppress the level of sphingolipids and thus present a cardiovascular protective effect. As for glycerophospholipids, including PC, PI, and PS, accumulating evidence showed that glycerophospholipids promoted the progression of cardiovascular diseases (69, 70). Studies showed that PC was positively associated with the prevalence of CAD (71), while moderate alcohol consumption was found to reduce the level of PC (25). Consistently, in our study, we found a decrease of PC (LPP1438, HMDB0008182) in MD-CAD when compared with HD-CAD and ND-CAD, suggesting that moderate alcohol consumption may reduce PC and lead to better cardiovascular health. In addition, it was reported that, in patients with hypertension, PI was found positively related to systolic blood pressure (SBP) (72). What is more, researchers found that alcohol-treated rats exhibited a decrease in PI and PS than control (25). Consistently, in our study, PI (LPN 207, LPN208, both reduced in MD-CAD) was positively correlated with CK-MB, a biomarker for myocardial injury. Overall, the reduction of glycerophospholipids associated with moderate alcohol consumption tended to be beneficial for cardiovascular health.

In addition to sphingolipids and glycerophospholipids, several other metabolites also caught our attention. Firstly, it was worth mentioning that 2-phenylacetamide (PLP4198, reduced in MD-CAD) positively correlated with *Bifidobacterium longum* (OTU 12, depleted in MD-CAD) (*P* < 0.029, Rho = 0.302) and uric acid (UA) (*P* = 0.003, Rho = 0.409). A high concentration of UA was associated with cardiovascular diseases (73). It was reported that 2-phenylacetamide (PLP4198) was the intermediate product in the bacteria fermentation of aromatic amino acid phenylalanine (Phe) into phenylacetic acid (PAA) (74). The downstream product of PAA in the host is PAGln, which acts via adrenergic receptors and increases the risk of thrombosis and CAD (18). Thus, we may infer that the reduction of 2-phenylacetamide (PLP4198) in MD-CAD may be associated with relevant bacteria, and thus improve the health of the cardiovascular system. Secondly, mevalonic acid (PLP5058, depleted in MD-CAD) was the key intermediate product in terpenoid backbone synthesis catalyzed by key enzyme 3-hydroxy-3-methyl glutaryl coenzyme A (HMG-CoA) reductase. Wang et al. observed a considerable elevation of mevalonic acid in hyperlipidemic rats and a decrease after lipid-lowering therapy with a folk medicine in China (75). As for the key enzyme in terpenoid backbone synthesis, genomic and phylogenetic analysis revealed that multiple bacteria had the similar HMG-CoA reductase as in human beings (76). According to the results of our study, we speculated that moderate drinking might inhibit cholesterol synthesis by restraining the proliferation of bacteria with the ability to synthesize mevalonic acid. Thirdly, 1-methylhistamine (PLP6139, depleted in MD-CAD) was a biomarker of allergic response, which was the main catabolite of histamine. Depleted 1-methylhistamine concentrations were observed in moderate drinkers when compared with heavy drinkers (77), and increased 1-methylhistamine was found in alcohol-preferring rats (78). Besides, 1-methylhistamine was a vital cardiac substrate that activated monoamine oxidase and induced reactive oxygen species production in the heart during oxidative stress (79). Our result was consistent with these findings and indicated that the reduction of 1-methylhistamine (PLP6139, depleted in MD-CAD) in moderate drinkers may play a role in the amelioration of allergic response and oxidative stress, owing to moderate alcohol consumption. In addition, 12-HPEPE (PLP2108, increased in MD-CAD) drew our special attention. An early study *in vitro* confirmed the potent platelet aggregation inhibitor 12-HPEPE could inhibit platelet aggregation and serotonin release (80). The rise of 12-HPEPE observed in MD-CAD may suggest a positive effect on the progression of atherosclerotic plaque.

Since several metabolites were microbiome derived, we paid attention to gut microbiome alterations in MD-CAD. The taxonomic features of MD-CAD resembled that of HC, and several gut microbes in MD-CAD altered significantly when compared with HD-CAD and ND-CAD. *Oscillibacter valericigenes* (OTU 57, elevated in MD-CAD) caught our special attention. It was reported that *Oscillibacter valericigenes* was depleted in people with Crohn's disease (81), and the decrease of the *Oscillibacter* genus may promote inflammation (82). Furthermore, genus *Oscillibacter* was reported to be able to produce butyric acid (83) and valeric acid (84). Butyric acid and valeric acid were both well-known beneficial substances for gut health. Thus, *Oscillibacter valericigenes* was considered a beneficial species. Our results indicated that *Oscillibacter valericigenes* (OTU 57, elevated in MD-CAD) may be promoted by alcohol and thus be beneficial to cardiovascular health. The relative abundance of *Enterococcus villorum* (OTU 19) was reduced in MD-CAD when compared with HD-CAD and ND-CAD. It was reported that *Enterococcus faecalis* was identified as a cause of hepatocyte death and liver injury (85). Since *Enterococcus villorum* (OTU 19) belonged to the same genus as *Enterococcus faecalis*, we suspected that the depletion may be beneficial to the liver. Our result indicated that moderate alcohol consumption may possibly promote potentially beneficial bacteria and may inhibit potentially pathogenic bacteria.

In addition, the multi-omics correlation revealed that moderate alcohol consumption was beneficial for cardiovascular health possibly by affecting the gut microbiome and serum metabolome. In our study, by moderate alcohol consumption, potential beneficial gut microbes (such as *Oscillibacter valericigenes* OTU 57 and *Paraprevotella clara* OTU 95) presented the highest abundance in MD-CAD among three disease groups, while potential pathogenic gut microbes (such as *Bacteroides ovatus* OTU 135 and *Bacteroides coprocola* OTU 14) showed the lowest abundance in MD-CAD when compared with ND-CAD and HD-CAD. Furthermore, the elevation of *Oscillibacter valericigenes* OTU 57 and *Paraprevotella clara* OTU 95 were both negatively associated with sphingolipids and glycerophospholipids (such as LPP138, LPP863, and LPP911), which were known to promote the progression of cardiovascular diseases (69, 70) by activating the proliferation and migration of vascular smooth muscle cells (68). Moreover, the depletion of potential sphingolipids and glycerophospholipids was positively associated with clinical indexes that indicate the severity of disease, such as Gensini score, CK-MB, FFA, and UA.

In a word, we came up with a new concept of “alcohol modulation through gut microbiome” in human cardiovascular health. It was found that alcohol consumption impacted many diseases causally, including ischemic heart disease, hypertensive heart disease, and stroke (86). We speculated that a moderate amount of alcohol consumption may modulate the intestinal microecology and serum metabolites, which may play an important role in the amelioration of CAD. Alcohol consumption should be taken into considerations as an important modulator in the cardiovascular system. However, there is still a need for functional validation and exploration studies to verify the cause-and-effect relationship in the future.

Several limitations of the study need to be acknowledged. First, although the clinical features of our cohort were consistent with common patients with CAD, the sample size was relatively small. Second, the study population only included men; hence, it was uncertain whether the finding could apply to women with CAD as well. Third, untargeted metabolomics had limited accuracy in the annotation of serum metabolites. Future work needs to be done to establish the exact relationship between moderate drinking and these alterations in serum metabolites and gut microbiota.

## Conclusions

In general, our study provided a novel insight into the effect of moderate alcohol consumption on cardiovascular health by affecting gut microbiota and serum metabolome. The impact on metabolites and microbiota in patients with CAD with moderate drinking seems to be separated from those in patients with CAD with heavy drinking or non-drinking. Drinking moderately may have more positive effects on the metabolic profiles and commensal flora of patients with CAD, which may explain how moderate drinking affects cardiovascular health.

## Data Availability Statement

The dataset supporting the results of this article has been deposited in the Sequence Read Archive under BioProject accession code SRP167862.

## Ethics Statement

The studies involving human participants were reviewed and approved by the Ethics Review Board at the Peking Union Medical College Hospital. The patients/participants provided their written informed consent to participate in this study.

## Author Contributions

XZ, RZ, and XH conceived and designed the study and wrote the manuscript. RZ and HL contributed to the bioinformatics analysis and made the tables and figures. YF and YS conducted the literature search. HL contributed to the collections of samples and data acquisition. XH and SZ critically revised the manuscript. All authors contributed to the article and approved the submitted version.

## Funding

This work was supported by the Beijing Natural Science Foundation (Grant No. 7202152), the National Natural Science Foundation of China (Grant No. 81670329 and 81974183), the CAMS Innovation Fund for Medical Sciences (CIFMS) (Grant No. 2017-I2M-2-002, 2016-I2M-1-002, and 2019-I2M-1-001), and the National Key Research and Development Program of China (Grant No. 2016YFC0901502).

## Conflict of Interest

The authors declare that the research was conducted in the absence of any commercial or financial relationships that could be construed as a potential conflict of interest. The reviewer WL declared a shared affiliation with all authors to the handling editor at the time of the review.

## Publisher's Note

All claims expressed in this article are solely those of the authors and do not necessarily represent those of their affiliated organizations, or those of the publisher, the editors and the reviewers. Any product that may be evaluated in this article, or claim that may be made by its manufacturer, is not guaranteed or endorsed by the publisher.

## Abbreviations

CAD: coronary artery disease
HDL-C: high-density lipoprotein cholesterol
SCFA: short-chain fatty acids
TMAO: trimethylamine N-oxide
PAGln: phenylacetylglutamine
SM: sphingomyelin
PUMCH: Peking Union Medical College Hospital
LC-MS: liquid chromatography-mass spectrometry
PLS-DA: partial least squares discriminant analysis
VIP: variable importance in the projection
OTU: operational taxonomic unit
PCoA: principal coordinate analysis
CPCoA: constrained principal coordinate analysis
PICRUSt2: Phylogenetic Investigation of Communities by Reconstruction of Unobserved States 2
BMI: body mass index
SCAD: stable coronary artery disease
UA: unstable angina
MI: myocardial infarction
HTN: hypertension
T2DM: type 2 diabetes mellitus
HLP: hyperlipidemia
FLD: fatty liver disease
CK-MB: creatine kinase-MB
TC: total cholesterol
TG: triacylglycerol
LDL-C: low-density lipoprotein cholesterol
ALT: alanine aminotransferase
PI: phosphatidylinositol
PC: phosphatidylcholine
PS: phosphatidylserine
cTnI: cardiac troponin I
Hs-CRP: high-sensitivity C-reactive protein
S1P: sphingosine-1-phosphate
SBP: systolic blood pressure
UA: uric acid
Phe: phenylalanine
PAA: phenylacetic acid
HMG-CoA: 3-hydroxy-3-methyl glutaryl coenzyme A
12-HPEPE: 12S-hydroperoxy-eicosapentaenoic acid
HTN drug: antihypertensive drugs
OAD: oral hypoglycemic drugs
AST: aspartate aminotransferase
CK: creatine kinase
ApoB: apolipoprotein B
ApoA1: apolipoprotein A1
FFA: free fatty acid
Lpa: lipoprotein (a)
Cr: creatine
Glu: fasting blood glucose
BUN: blood urea nitrogen
no. vessel: number of stenosed vessels.
