# Supplementary material for: The Effects of Moderate Alcohol Consumption on Circulating Metabolites and Gut Microbiota in Patients With Coronary Artery Disease
Source: Front Cardiovasc Med. 2021 Nov 2;8:767692. doi: 10.3389/fcvm.2021.767692 (PMC8593214; doi:10.3389/fcvm.2021.767692)
Supplement: Supplementary file 2 [file Image_1.pdf]

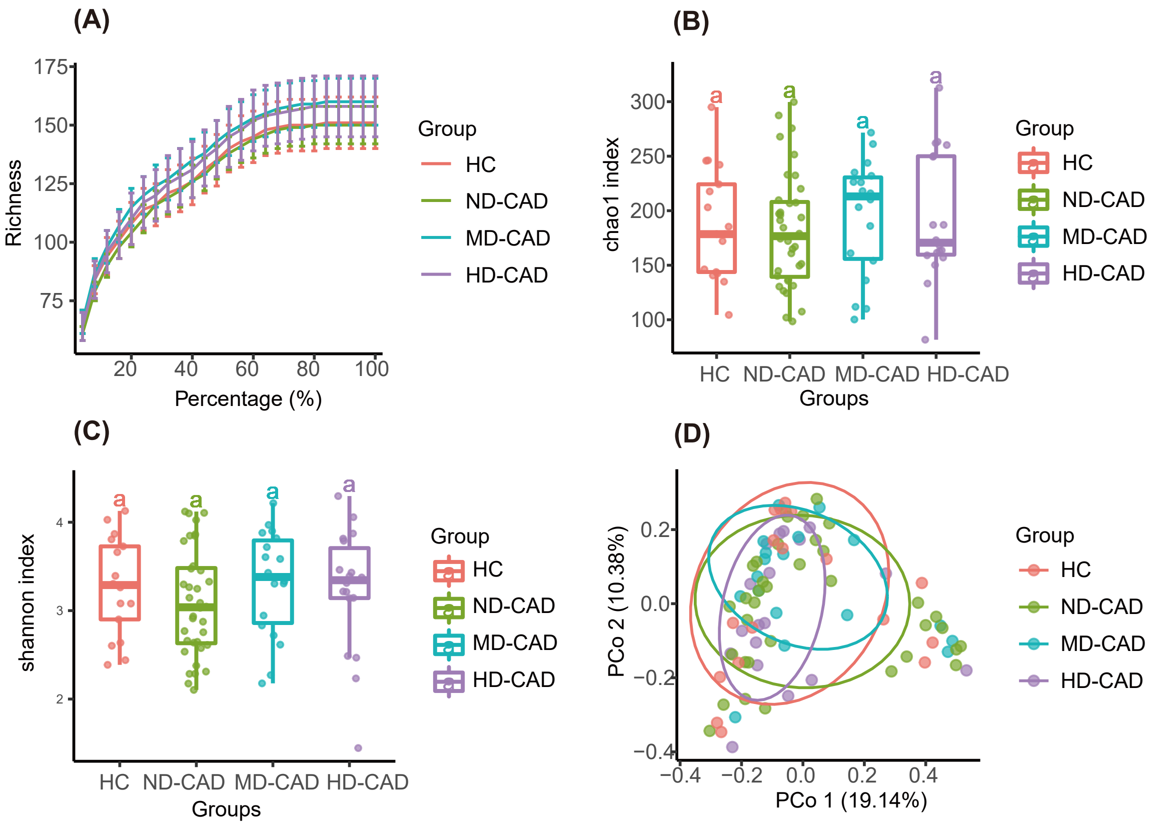

Figure S1| (A) Rarefaction curves. (B and C) Alpha diversity analyzed by chao1 index (B) and Shannon's index (C) (D) PCoA score plot of all samples by Bray-Curtis distances. (HC vs. ND-CAD: *P*= 0.71; HC vs. MD-CAD: *P*= 0.75; HC vs. HD-CAD: *P*= 0.36; ND-CAD vs. MD-CAD: *P*=0.72, ND-CAD vs. HD-CAD: *P*=0.24, MD-CAD vs. HD-CAD: *P*=0.27, Adonis test). ns: not specific. **P*<0.05, Wilcoxon rank-sum test.
