# Supplementary material for: The Effects of Moderate Alcohol Consumption on Circulating Metabolites and Gut Microbiota in Patients With Coronary Artery Disease
Source: Front Cardiovasc Med. 2021 Nov 2;8:767692. doi: 10.3389/fcvm.2021.767692 (PMC8593214; doi:10.3389/fcvm.2021.767692)
Supplement: Supplementary file 3 [file Image_2.pdf]

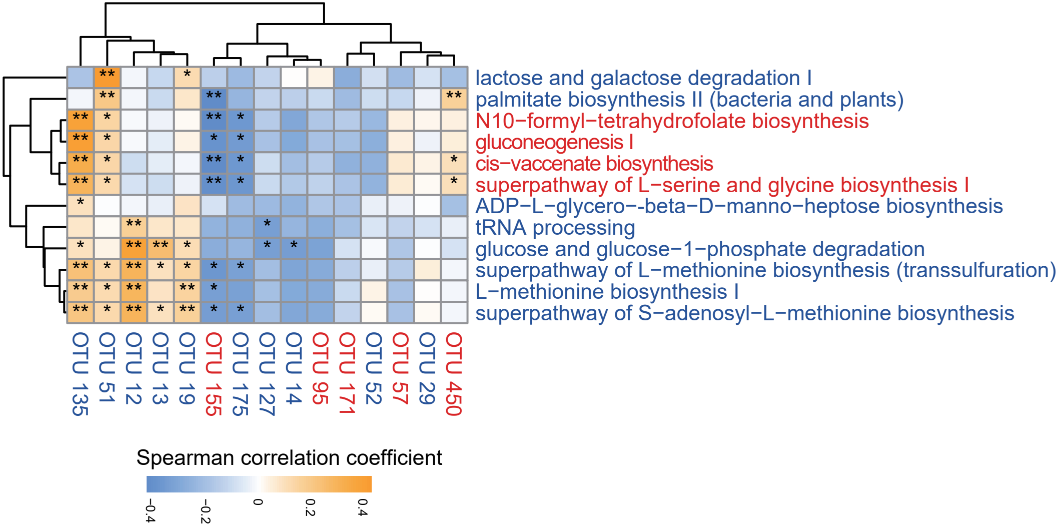

Figure S2| Spearman correlations between key OTUs associated with moderate alcohol consumption and pathways. The pathways predicted by PICRUSt2 were significantly different between moderate drinkers with CAD and non-drinkers with CAD. The OTUs are highlighted in red (enriched in MD-CAD) or blue (depleted in MD-CAD). The pathways are highlighted in red (upregulated in MD-CAD) or blue (downregulated in MD-CAD). *P < 0.05, **P < 0.01.
